# GPX3 and GSTT1 as biomarkers related to oxidative stress during renal ischemia reperfusion injuries and their relationship with immune infiltration

**DOI:** 10.3389/fimmu.2023.1136146

**Published:** 2023-03-22

**Authors:** Jun Pei, Xiaomao Tian, Chengjun Yu, Jin Luo, Jie Zhang, Yi Hua, Guanghui Wei

**Affiliations:** ^1^ Department of Urology, Children’s Hospital of Chongqing Medical University, Chongqing, China; ^2^ Ministry of Education Key Laboratory of Child Development and Disorders, Chongqing Key Laboratory of Pediatrics, National Clinical Research Center for Child Health and Disorders, China International Science and Technology Cooperation Base of Child Development and Critical Disorders, Children’s Hospital of Chongqing Medical University, Chongqing, China; ^3^ Chongqing Key Laboratory of Children Urogenital Development and Tissue Engineering, Chongqing, China

**Keywords:** oxidative stress, immune infiltration, ischemia-reperfusion injury, kidney, GPX3

## Abstract

**Background:**

Renal ischemia reperfusion injuries (IRIs) are very common in clinical diagnoses and treatments, which are a common cause of impaired renal functions, worsening pathological damage, affecting disease progression and hindering recovery. Renal IRIs are an inflammatory disease mediated by the adaptive and innate immune system. There is a complex interaction between oxidative stress and immune cell infiltration. Therefore, we aimed to determine biomarkers associated with oxidative stress during renal IRIs and their relationship with immune cell infiltration.

**Method:**

A differential gene expression analysis was made based on the GSE148420 dataset from the NCBI Gene Expression Comprehensive Database (GEO) combined with 92 oxidative-stress (OS)-related genes identified in the Molecular Signatures Database. Then we identified differentially-expressed genes (DEOSGs) associated with oxidative stress, which were used for gene ontology (GO) and a Kyoto Encyclopedia of Genomes (KEGG) enrichment analysis. At the same time, we used PPI protein interaction networks and Lasso regression analysis to identify key genes, which were verified by the validation sets GSE58438 and GSE71647, as well as Western Blot detection on rat renal IRI models. At the same time, PAS staining, HE staining and immunohistochemistry were used to detect tissue damage and expression of markers related to oxidative stress during renal ischemia-reperfusion. Single-gene enrichment analysis (GSEA) was used to further clarify the underlying biological functions of key genes. Cibersort was used to analyze the immune cell infiltration during renal IRI and the correlation of key genes with immune cells. At the same time, we constructed a network of transcription-factor (TF)-Hub genes and miRNA-Hub genes. DGIDB was used to predict drugs and molecular compounds that might interact with the Hub genes.

**Results:**

Compared with the control group, a total of 5456 differential genes (DEGs) were measured in the renal IRI group, 2486 of which were upregulated and 2970 were down-regulated. Among them, we found 30 DEGs (DEOSGs) associated with oxidative stress. The results of GO and KEGG enrichment analysis showed that these DEOSGs were mainly enriched in glutathione metabolism, the response to oxidative stress stimulation, the regulation of T cell activation and apoptosis signaling pathways. Through a protein interaction network (PPI) and a LASSO regression analysis, a total of two Hub genes were identified, namely GPX3 and GSTT1, which were validated through external validation sets and animal experiments. Through pathological methods, we found that the pathological damage of renal tissue and the expression of oxidative stress markers increased after renal ischemia-reperfusion. The results of GSEA showed that the Hub genes were related to oxidative stress pathways, apoptosis signaling pathways and immune-response-related signaling pathways. An immunoinfiltration correlation analysis showed that genes GPX3 and GSTT1 were significantly positively correlated with plasma cells and macrophage M0, while were negatively correlated with monocytes and macrophages M1 and M2. Using the Strust, Starbase and DGIDB database, we predicted that 81 transcription factors, 49 miRNAs and 13 drug or molecular compounds might interact with the Hub genes.

**Conclusion:**

Through a comprehensive analysis of gene expression, our findings may provide new potential biomarkers for the pathogenesis of renal IRIs and a reliable basis for its early diagnosis as well as treatment.

## 1 Introduction

Ischemia reperfusion injuries (IRIs) refer to the occurrence of blood supply recovery and organ reperfusion after a period of blood blockage flow, resulting in the aggravation of corresponding ischemic hypoxia injuries. IRIs are very common in the process of clinical diagnosis and treatment, such as myocardial infarction, stroke, trauma, severe infections and organ transplantation, etc. Ischemia reperfusion usually leads to the exacerbation of histopathological injuries, affecting progression and delaying recovery. Kidney transplantation is currently an effective clinical treatment for end-stage renal diseases (1). During kidney acquisition and transplantation, the kidney will inevitably suffer ischemia reperfusion damage, which can cause a delayed recovery of renal functions in the early stages after transplantation, or the loss of the graft function at worst, and may even cause graft rejection (2). Renal ischemia reperfusion injuries (RIRIs) have been found to be an important factor for the decline and delayed recovery of transplanted kidney functions after kidney transplantation (3), which is associated with an acute rejection and a prolonged hospital stay (4). Therefore, understanding and responding to RIRIs are an extremely important part of clinical diagnosis and postoperative management.

Oxidative stress is the initiating factor of reperfusion injuries, and the production as well as scavengation of reactive oxygen species (ROS) is in a dynamic equilibrium under normal physiological conditions (5). When this equilibrium is disrupted, oxidative stress occurs, which subsequently promotes the occurrence of inflammatory responses, the dysregulation of autophagy and an increased apoptosis. Current theories suggest that oxidative stress is closely related to RIRIs (6). When IRIs occur in the kidneys, the tissues are stimulated by oxidative stress damage, the inflammatory cluster reaction is activated, and a variety of inflammation-related factors, such as tumor necrosis factors (TNFs), interleukin-6 (IL-6) and cytokines, chemokines, etc., are activated and released (7). RIRIs also lead to vascular endothelial cell damage, an increased vascular permeability and increased cell surface adhesion molecules, induce inflammatory cell infiltration, further promote the release of inflammatory factors, accelerate cell necrosis apoptosis and aggravate tissue damage (7). In previous studies, we have found that when kidneys suffer from IRIs, oxidative stress can be reduced through normobaric hyperoxia intervention, which increases antioxidant production and reduces tissue damage caused by renal ischemia reperfusion (8). At the same time, a large number of studies have proved that when kidneys suffer from IRIs, the oxidative stress damage of the kidneys can be reduced through a timely intervention of antioxidant substances, which has a certain protective effect on renal functions (9, 10). It can be seen that measures against oxidative stress are one of the important directions for the prevention and treatment of RIRIs.

In previous studies, it has been found that IRIs, as an acute tissue injury lesion, can stimulate and excite the innate immune system, activate nonspecific immune cells, such as macrophages, neutrophils and monocytes, etc., as well as produce corresponding immune responses (11, 12). IRIs are an inflammatory disease mediated by the adaptive and innate immune system (11). Studies have pointed out that in the process of IRIs, the innate immune response acts as the first line of defense through neutrophils, macrophages, NK cells and T cells, etc. (11). The activation of the innate immune system occurs within minutes, but the adaptive immune responses occur a few days after the IRIs. In RIRIs, T-cell-antigen-specific or nonspecific responses are thought to play a crucial role. Snelgrove et al. used T-cell-deficient mice and overexpression to metastasize T cells, finding that T cells played an important role in RIRIs (13). Kinsey et al. found that the local inflammatory response of kidneys was weakened due to CD4+ T cell supplementation in mouse RIRI models, which had a protective effect on RIRI damage (14). These studies provide new insights into the role of immunomodulation in RIRIs. However, the role of oxidative stress processes in RIRIs and their relationship with immune regulation remain unclear.

In summary, in this study, we performed a systematic bioinformatic analysis based on differentially-expressed oxidative-stress-related genes, so as to identify key genes involved in the IRI process in the kidneys and analyze their associations with immune infiltration.

## 2 Materials and methods

### 2.1 Dataset sources and processing

To identify potential molecular markers associated with oxidative stress during RIRIs, in the first step, we searched in the GEO database (Gene Expression Omnibus, http://www.ncbi.nlm.nih.gov/geo) using the keywords “Renal and IRI” and selected the mRNA expression profiles from three datasets for this study, namely Data GSE148420, using the GPL14746 platform (Agilent-028282 whole mouse genome microarray), which included 4 IRI groups and 4 Sham groups; Data GSE58438, using the GPL11534 platform (Affymetrix Rat Gene 1.1 ST microarray), which included 9 IRI groups and 5 Sham groups; and Data GSE71647, using the GPL7202 platform (Agilent-014868 whole mouse genome microarray), which included 4 IRI groups and 4 Sham groups, To ensure homology of the analytical data, we perform homology gene conversion *via* the “Homologous Genes” toolbox in the online tool Bioinformatics (http://www.bioinformatics.com.cn). The above three datasets were normalized using the “Limma” R package. At the same time, we used GSE148420 as the training set, and GSE58438 and GSE71647 as the external validation sets. In the second step, we obtained a total of 92 sets of genes (OS) associated with oxidative stress from the Molecular Signatures Database.

### 2.2 Identification and analysis of differential genes associated with oxidative stress (DEOSGs)

Through the Limma R software package, the classical Bayesian algorithm was used to calculate the differentially-expressed genes (DEGs) in the dataset GSE148420. The absolute value of Log2Fold > 1.0 and adj.P<0.05 were used as significance indicators. The Hiplot (https://hiplot.org) visual biomedical online analysis toolbox was used to construct DEG heat maps and volcano maps (15). The intersection of DEGs and oxidative-stress-related genes (OSs) obtained in the Molecular Signatures Database is defined as oxidative-stress-related differential genes (DEOSGs) by us. To reveal the underlying biological functions and underlying mechanisms of DEOSGs, we used the Hiplot (https://hiplot.org) online analysis tool for gene ontology (GO) and Kyoto Encyclopedia of Gene and Genes (KEGG) enrichment analysis of DEOSGs (15). Hiplot is a visualization application equipped with 240+ pieces of biomedical data, covering basic statistics, multiomics, regression, clustering, dimensionality reduction, meta-analysis, survival analysis, risk modeling and other functions (15). GO enrichment analysis includes biological processes (BPs), cellular components (CCs) and molecular functions (MFs); adj. P<0.05 is the threshold for screening the main enrichment functions and pathways of DEOSGs.

### 2.3 Identification of hub genes based on protein-protein interaction (PPI) networks and LASSO regression in machine learning

To further explore the interactions among DEOSGs, we constructed a PPI network of DEOSGs or protein interactions using the online biological resource database String (http://www.string-db.org/). We used the confidence score of 0.4 as a cut-off value to visualize the PPI network of DEOSGs using Cytoscape software. We identified key genes in the PPI network of DEOSGs using three evaluation metrics from the CytoNCA plug-in (Degree Centrality (DC), Betweenness Centrality (BC) and Closeness Centrality (CC)). In addition, in order to obtain the target key genes more accurately, we used the “glmnet” package for Lasso regression algorithm to re-identify them. Subsequently, the key genes screened through the PPI protein interaction network and the LASSO regression algorithm were intersected, and the intersection gene was defined as the most valuable oxidative-stress-related Hub gene for RIRIs. Subsequently, we performed a ROC curve analysis of the Hub gene using a Hiplot (https://hiplot.org) visual biomedical online analysis toolbox to verify its accuracy, which was considered diagnostic with AUC>0.7 (16). At the same time, we also performed a statistical analysis on the expression of the Hub gene in IRI group and Sham group, where P<0.05 was considered to be statistically significant.

### 2.4 Establishment of animal models

We enrolled a total of 10 adult male Sprague-Dawley (SD) rats from the Laboratory Animal Center of Chongqing Medical University (SYXK[YU] 2022-0016, Chongqing, China), weighing between 250-280 g, all of which were raised under the same conditions. All rats underwent right kidney removal and were randomly divided into two groups, the IRI group and Sham group. In the IRI group, the left renal pedicle was bluntly separated, and non-invasive vascular clamping was performed for 45min, and the color of their kidneys changed from bright red to purple-black, indicating that successful ischemia. After releasing the vascular clamp, it was observed that the color of the kidneys gradually returned to bright red, indicating a successful reperfusion. In contrast, the Sham group only underwent blunt isolation of the left renal pedicle without ischemia treatment. After 24h, both groups of rats died suddenly under anesthesia, and their left kidneys were collected for Western Blot detection to determine the expression of Hub genes. Furthermore, pathological detection was performed to evaluate kidney damage in the two groups. The animal experiments were approved by the Animal Ethics Committee of the Children’s Hospital of Chongqing Medical University (IACUC Issue No: CHCMU-IACUC20220429002).

### 2.5 Verification of the hub genes

To further determine the diagnostic value of the Hub genes, first of all, we performed a ROC curve analysis based on the external validation sets GSE58438 and GSE71647 to verify their accuracy, and the Hub genes with the same AUC>0.7 were considered diagnostic. At the same time, we performed a statistical analysis on the expression of the Hub genes between the IRI group and the Sham group in the external validation set, and P<0.05 was considered statistically significant. Secondly, we used the Western Blot method to detect the expression of the Hub genes between the RIRI group and the Sham group; Both GPX3 and GSTT1 antibodies were purchased from Wuhan ABclonal Biotechnology Co., Ltd. (A12856, A1287). The kidney tissues of each group of rats were added to the corresponding lysis buffer, the tissues were homogenized, centrifuged and the supernatant was collected. Protein concentration was determined according to the BCA method. Corresponding tissue protein was taken for SDS-PAGE electrophoresis, followed by membrane transfer. The primary antibodies (both at 1:2000 dilution) was incubated overnight at 4°C. On the following day, the secondary antibodywas incubated at room temperature for 1 hour then washed with TBST again. The band of interest was semi-quantitatively analyzed by calculating the ratio of the band of the protein of interest to the band of the β-actin protein. P<0.05 was considered significant.

### 2.6 Detection of histopathological damage to rat kidney

The left kidney of the rat was fixed in paraformaldehyde solution, then HE staining and PAS staining were performed after routine dehydration, transparency, embedding and sectioning. Slides were observed under light microscopy. The tubular injury score was rated based on the Paller method (17). The evaluation method was to observe the renal cortex under 400x microscopy, randomly select 10 renal tubules in each high-magnification field, and randomly select 10 fields for a total of 100 renal tubules for each specimen. The tubular injury score was determined according to the following criteria: 1 point for vacuolar degeneration of tubular epithelial cells; Renal tubular conspiration with cell flattening scored 1 point; 1 point for brush edge damage and 2 points for shedding; Epithelial cell necrosis scored 2 points; 2 points for tube formation; Normal tubules score 0. As long as one of the above pathological changes occurred in the selected renal tubules, the corresponding score was recorded. If two or more pathological changes occur in the same renal tubule, the score was scored based on the highest injury score. The final score for each specimen was the total score of 100 tubules.

Kim-1 and NGAL are often used as markers of tubular injury to assess tubular epithelial cell damage. We performed immunohistochemical staining using the EnVision two-step method, strictly following the instruction. The antibodies were diluted 1:200. Both Kim-1 and NGAL were positively expressed in the cytoplasm of tubular epithelial cells. Image J software was used for semi-quantitative protein analysis of Kim-1 and NGAL. The specific operation is as follows: Under a 200x optical microscope, each specimen randomly selected 5 non-overlapping fields of view in the renal cortex and measured their average optical density (OD). The average OD value of the 5 fields of view was the OD value of the specimen, all under the same optical conditions.

### 2.7 Detection of markers of oxidative stress damage

Hypoxia is known contributing factor in the development of acute kidney injury (AKI), especially in the case of renal ischemia-reperfusion injury (RIRI). The expression of hypoxia-induced factor 1-α (HIF1-α) can clear ROS and regulate cell survival, exerting a protective effect on RIRI. Heme oxidase-1 (HO-1) is an essential antioxidant in the body and is often used to measure the reduction of ROS-induced damage during ischemia-reperfusion. We performed immunohistochemical staining using the EnVision two-step method, and the primary antibodies were diluted at 1:200, and both HIF1-α and HO-1 were positively expressed in the cytoplasm of renal tubular epithelial cells. Image J software was used for semi-quantitative analysis of HIF1-α and HO-1. The results were assessed as described in the previous paragraph.

### 2.8 Single-gene enrichment analysis (GSEA)

We performed a SGEA using the “Clusterproiler” (V3.16.1) R package to elucidate significant functional and pathway differences in the Hub genes between the IRI and Sham group. The number of gene set permutations per analysis is 1000 times. The criterion for a significant gene enrichment was P <0.05.

### 2.9 Correlation analysis between hub genes and immune cell infiltration

The proportion of 22 different types of immune cells in the sample GSE148420 of the training set was calculated through Cibersort (http://cibersort.stanford.edu/) (18). The expression level of the 22 types of immune cells in the IRI and Sham group was compared with the “VioPlot” software package. A Spearman-related analysis of infiltrating immune cells with Hub genes was calculated using “Corrplot” in R. We used lollipops to visually diagnose the correlations between the Hub genes and immune cells. P<0.05 was considered statistically significant.

### 2.10 Construction of a transcription factor (TF)-hub gene network and a miRNA-Hub gene network

Trrust (https://www.grnpedia.org/trrust/) is a reliable and intuitive tool for transcriptional regulatory networks (19), which can provide key TFs for multiple genes and regulatory information on interactions. We used the TRRUST database to predict TFs that regulated the Hub genes. The Starbase V2.0 database (http://starbase.sysu.edu.cn/) contains data from five databases: TargetScan, Pictar, Pita, Miranda/MirsVR and RNA22, in which the most comprehensive range of miRNA-lncRNAa, miRNA-pseudogene, miRNA-circRNA, miRNA-mRNA and protein-RNA interaction networks is built (20). We used the Starbase database to predict miRNAs that were targeted at and bound to the Hub genes. Finally, we mapped the regulatory network of TF-Hub genes and miRNA-Hub genes through Cytoscape software.

### 2.11 Potential drug identification of the hub genes

In the Drug-Gene Interactions Database (DGIdb, www.dgidb.org), information on drug-gene interactions and possible druggability of genes from 30 different sources are integrated, synthesized and normalized (21). We used the DGIdb database to predict drugs and molecular compounds that could interact with the Hub genes. Cytoscape software was used to visualize drug-gene interaction networks.

### 2.12 Statistical analysis

All bioinformatics analyses were performed using R software; Prism software (GraphPad software, La Jolla, CA) was used for statistical analyses of experimental data. **** represents P<0.0001, *** represents P<0.001, ** represents P<0.01, * represents P<0.05.

## 3 Results

### 3.1 Identification and enrichment analysis of DEOSGs

**Figure 1:**
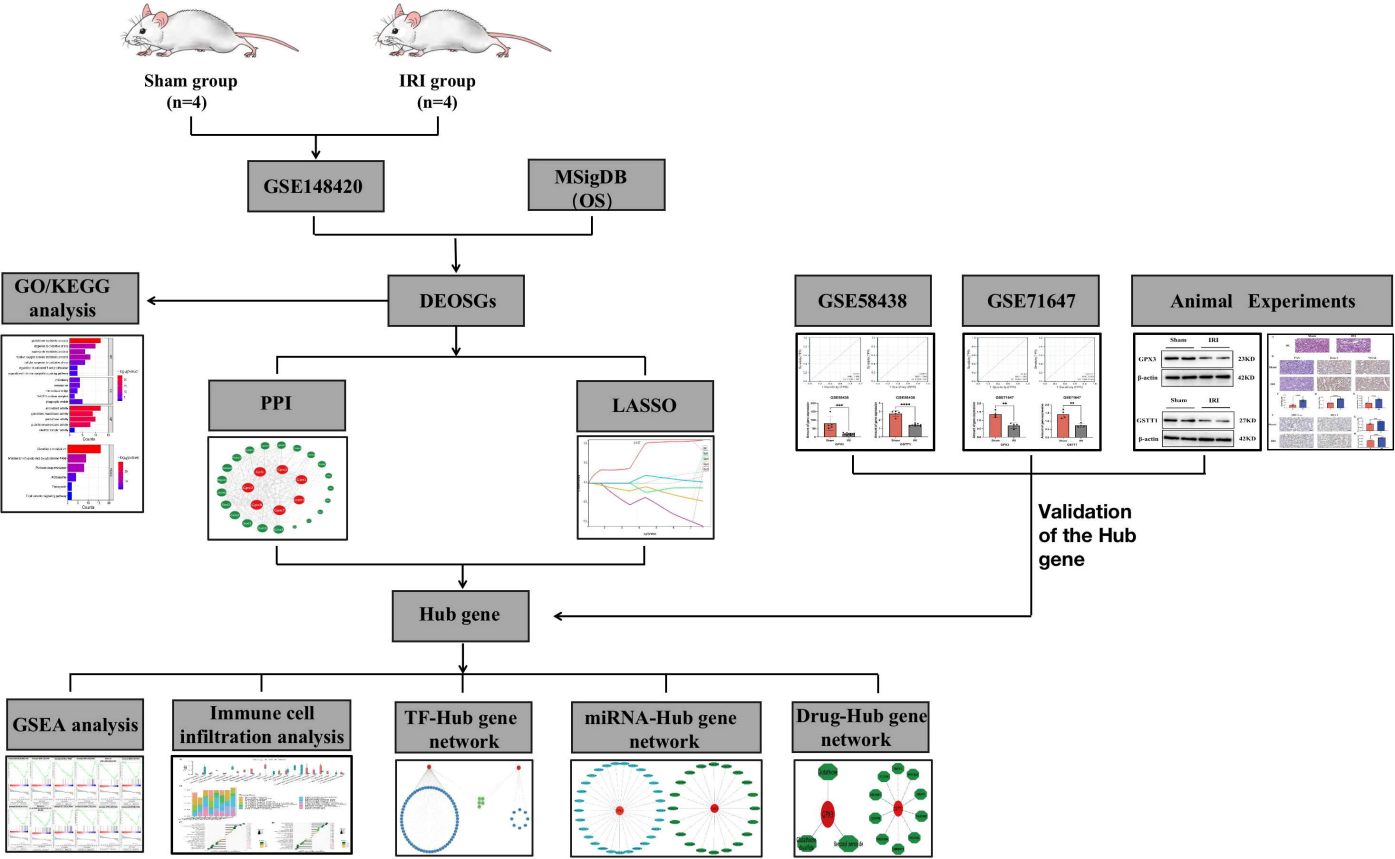
Research flowchart.

**Figure 2:**
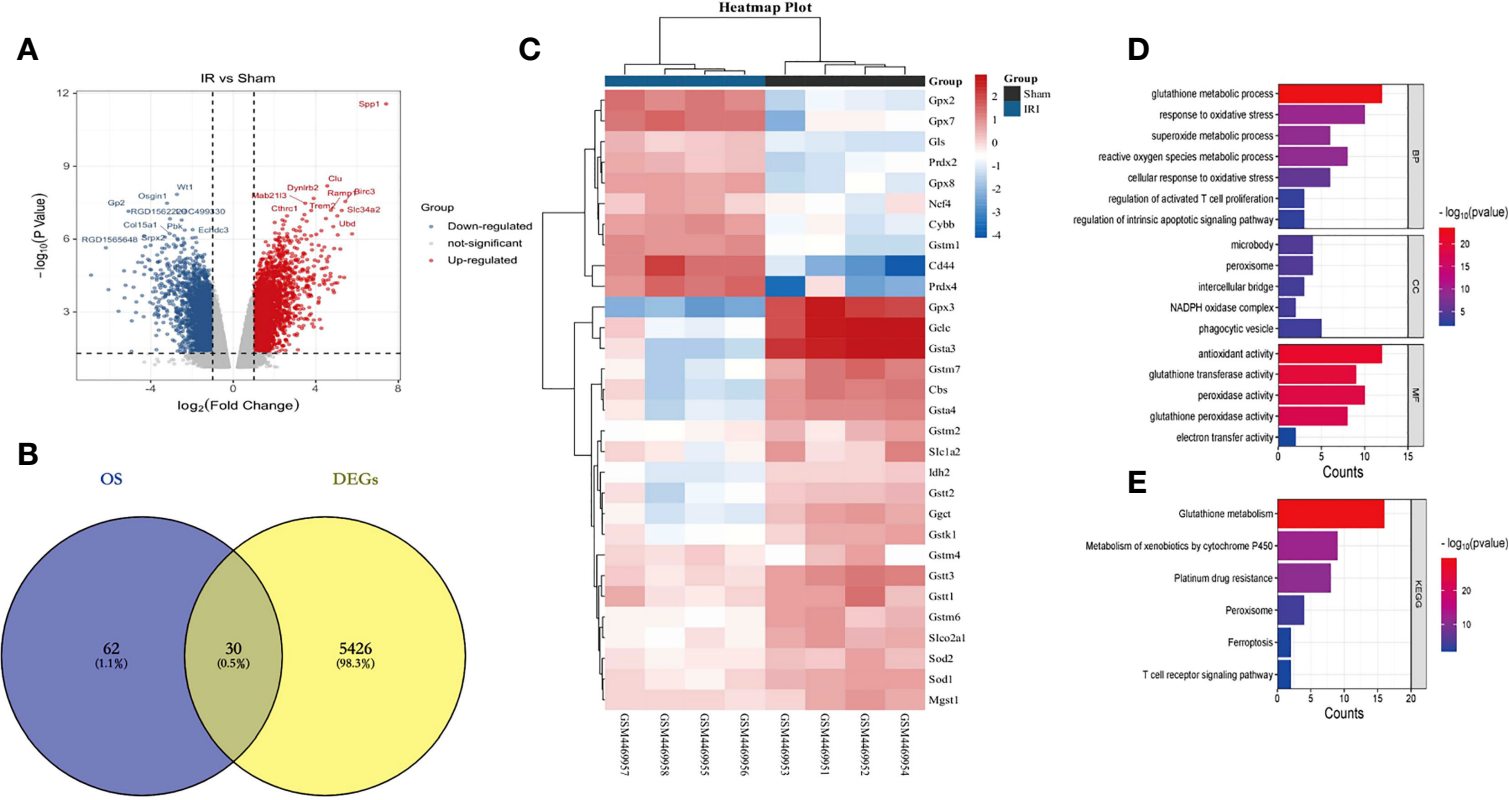
Identification and enrichment analysis of DEOSGs. **(A)** represents the GSE148420 dataset in the DEG volcano map; **(B)** represents the Veen plot of OS-related genes and DEGs in GSE148420; **(C)** represents the gene heatmap of DEOSGs; **(D)** represents the GO enrichment analysis plot of DEOSGs; **(E)** represents the KEGG enrichment analysis plot of DEOSGs.

A flowchart of this study is shown in **Figure 1**. In the training set GSE148420, a total of 5456 DEGs (Log2Fold absolute value > 1.0 and adj.P <0.05) were measured, of which 2486 were upregulated and 2970 were downregulated in the IRI group. The volcano map shows the distribution of those DEGs (**Figure 2A**). In order to further identify DEGs related to oxidative stress among differentially-expressed genes, we obtained a total of 92 genes (OS) related to oxidative stress from the Molecular Signatures Database, and obtained 30 genes after an intersection with DEG-related intersection genes, which we defined as DEOSGs (**Figure 2B**). The heat map shows that DEOSGs can be used to distinguish between the IRI and Sham group (**Figure 2C**).

To further determine the potential functions and pathways of DEOSGs, through a GO enrichment analysis, we found that in the BP, DEOSGs were mainly enriched in glutathione metabolism, responses to oxidative stress stimulationthe as well as regulation of T cell activation and apoptosis signaling pathways (**Figure 2D**). Among the CCs, DEOSGs are mainly enriched in peroxisomes, intercellular bridges and NADPH oxidase complexes (**Figure 2D**). In MFs, DEOSGs are mainly enriched in the activation of antioxidant enzymes, glutathione transferase and peroxidase (**Figure 2D**). After a KEGG pathway analysis, we found that DEOSGs were mainly enriched in glutathione metabolism, cytochrome-P450-mediated metabolism of harmful substances, peroxidase, ferrozoticosis and T cell receptor signaling pathways (**Figure 2E**).

### 3.2 Identification of hub genes based on PPI and LASSO regression

**Figure 3:**
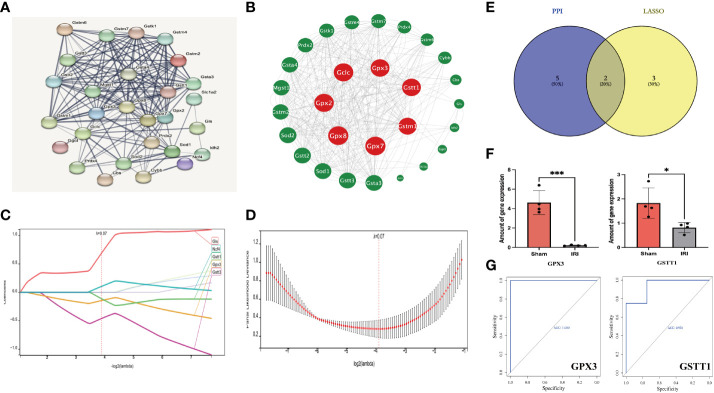
Identification of Hub genes based on PPI and LASSO regression analysis. **(A, B)** represents PPI protein interaction network analysis (using 3 evaluation indicators in the CytoNCA plugin: DC, BC and CC, red is the key gene for screening); **(C, D)** stands for LASSO regression analysis (5 key genes were finally obtained with a Lambda value of 0.0676); **(E)** represents the Veen plot of key gene intersection screened through PPI and Lasso regression analysis; **(F)** represents the ROC curve analysis plot of the Hub genes in the GSE148420 dataset; **(G)** represents the statistical plot of the expression of Hub genes between the IRI and Sham group in the GSE148420 dataset;. *** represents P<0.001, * represents P<0.05.

First of all, we input the 30 DEOSGs obtained above into the String database, screened the proteins that interacted with them (**Figure 3A**), and imported the results into Cytoscape software to build a PPI network (**Figure 3B**). Using the three evaluation indexes in the CytoNCA plug-in (Degree Centrality (DC), Betweenness Centrality (BC) and Closeness Centrality (CC)), the first 7 genes were identified as key genes, namely: GPX2, GPX3, GPX7, GPX8, GCLC, GSTT1 and GSTM1 (**Figure 3B**).

Secondly, in order to further improve the accuracy of key genes, we used Lasso regression analysis in machine learning algorithms to obtain 5 key genes when the Lambda value was set as 0.0676, namely GLS, GSTT3, NCF4, GSTT1 and GPX3 (**Figures 3C, D**). After intersecting the key genes of PPI with Lasso regression screening, the intersection genes: GSTT1 and GPX3, were finally determined as the most diagnostically-valuable Hub genes (**Figure 3E**).

Finally, in order to verify the accuracy of the Hub genes, we diagnosed the sensitivity and specificity of the predictive model through subject-based operating characteristic (ROC) curve analysis in the training set GSE148420, quantified with the area under the ROC curve (AUC), and the AUC of the Hub genes was greater than 0.7 (**Figure 3F**). The results of statistical analysis between the IRI and Sham group showed that P was less than 0.05 (**Figure 3G**). Therefore, we believe that the Hub genes GSTT1 and GPX3 may have some influence on the pathophysiological process of RIRIs.

### 3.3 Verification of the hub genes

**Figure 4:**
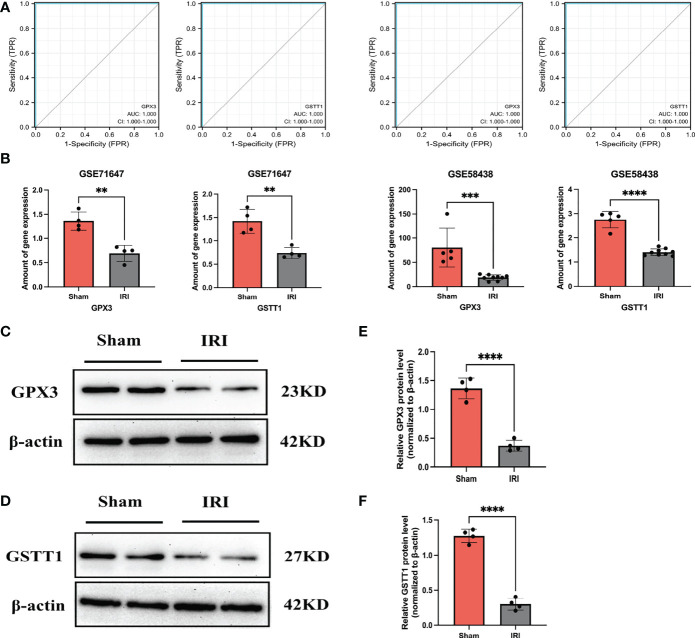
Validation of the Hub genes. **(A)** represents the ROC curve analysis plot of GSTT1 and GPX3 in the validation sets GSE58438 and GSE71647; **(B)** represents the expression statistics of GSTT1 and GPX3 in the Sham and IRI group in the validation sets GSE58438 and GSE71647; **(C, D)** represents the Western Blot protein band plot of GPX3 and GSTT1 in renal tissues in the RIRI model; **(E, F)** represents the Western Blot proteins of GPX3 and GSTT1 expressed statistically in the and IRI group. **** represents P<0.0001, *** represents P<0.001, ** represents P<0.01.

Through the above analysis process, we identified GSTT1 and GPX3 as possible biomarkers associated with oxidative stress during RIRIs. In order to further verify their accuracy, we first performed a ROC curve analysis on the expression of Hub genes in external validation sets GSE58438 and GSE71647, and found that AUC was greater than 0.7 (The AUC values for GPX3 and GSTT1 in the GSE71647 and GSE58438 datasets were both 1) (**Figure 4A**). At the same time, the expression of Hub genes in the IRI and Sham group was statistically analyzed, it was found that the expression trend of Hub genes in the validation sets GSE58438 and GSE71647 was the same as that in the training set GSE148420, and there was a statistical difference between their expression (P<0.05) (**Figure 4B**). Secondly, in the rat RIRI model, after a Western Blot detection of kidney tissues, we found that the expression of GSTT1 and GPX3 in the Sham group was significantly higher than that in the IRI group, and the difference was statistically significant (**Figures 4C–F**), which was consistent with our previous results. GSTT1 and GPX3 were further identified as biomarkers associated with oxidative stress in RIRIs.

### 3.4 Assessment of histopathological injury of the kidneys

**Figure 5:**
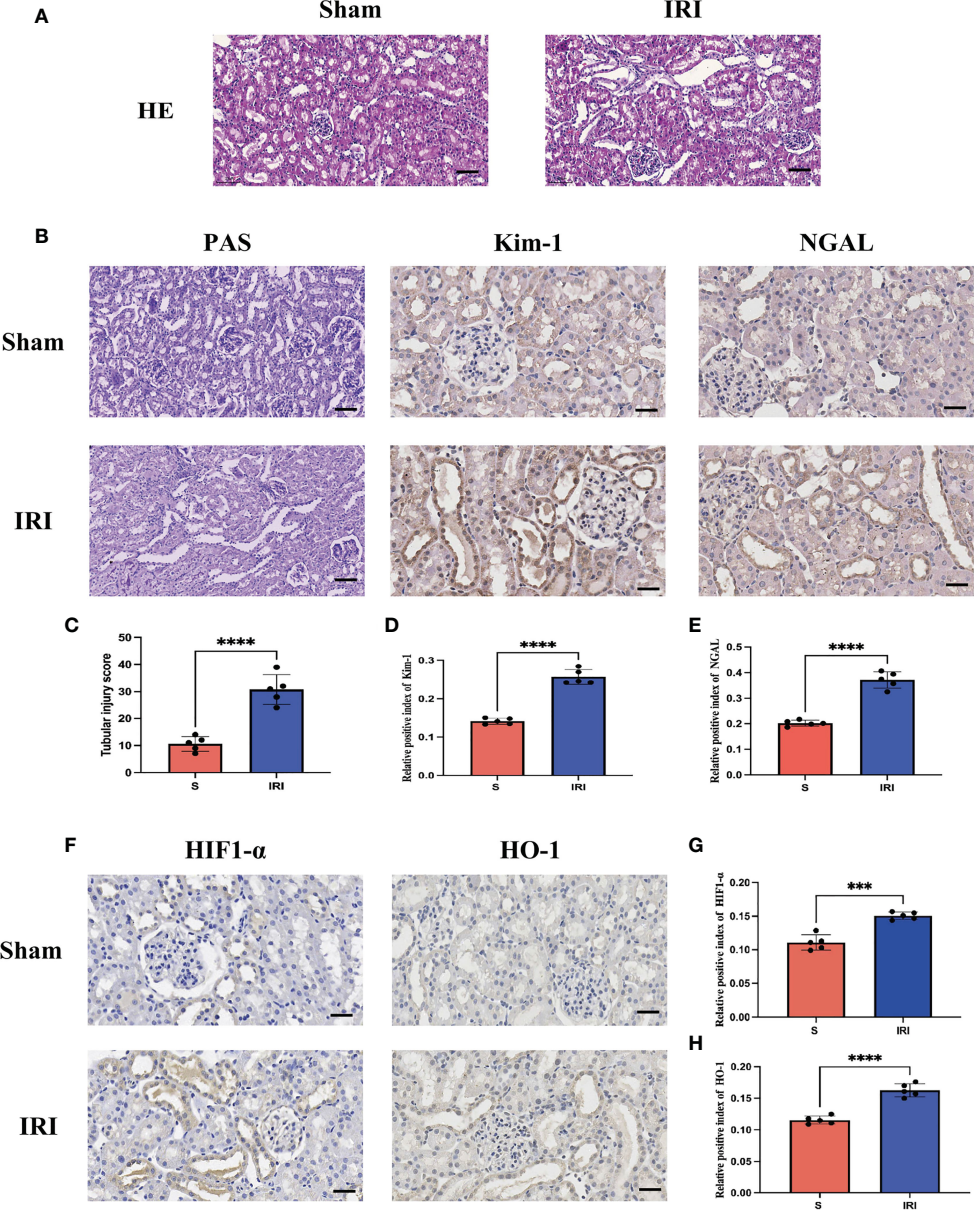
Evaluation of renal pathological damage and markers of oxidative stress. **(A)** represents the HE staining pattern of renal tissue in the rat renal ischemia-reperfusion injury model; **(B)** represents PAS staining of renal tissue in rat renal ischemia-reperfusion injury model and renal tubular injury markers Kim-1 and NGAL immunohistochemistry; **(C)** stands for tubular injury score chart; **(D, E)** represents the average optical density statistical plot of Kim-1 and NGAL immunohistochemical positive expression, respectively. **(F)** stands for oxidative stress markers HIF1-α and HO-1 immunohistochemistry; **(G, H)** represents the average optical density statistical plots of positive expression of HIF1-α and HO-1 immunohistochemistry, respectively. **** represents P<0.0001, *** represents P<0.001.

After evaluating the pathological damage caused by renal ischemia-reperfusion by HE staining, PAS staining and immunohistochemistry, we obeserved that the glomerular and renal tubular structures in the Sham group were normal under the light microscope, as shown in **Figures 5A, B**. In contrast, in the IRI group, the tubular structure was disordered, the renal tubule was significantly dilated, the tubular epithelial cells were necrotic and detached, and there were debris in the lumen. The tubular injury scores were significantly higher than in the IRI group compared to the Sham group (**Figure 5C**). Furthermore, the expression of renal tubular injury markers Kim-1 and NGAL was significantly increased in the IRI group compared to the Sham group (**Figures 5D, E**). These results are consistent with previous studies, indicating that renal histopathological damage is significantly aggravated when the kidneys are subjected to ischemia-reperfusion.

### 3.5 Evaluation of markers of oxidative stress

HIF1-α and HO-1 are often used as markers to assess oxidative stress levels during ischemia-reperfusion. Immunohistochemical detection of markers of oxidative stress, HIF1-α and HO-1 (**Figure 5F**), revealed that their expression was significantly increased in the IRI group compared to the Sham group, with a statistically significant difference (**Figures 5G, H**). It has been demonstrated that during renal ischemia-reperfusion injury, the oxidative stress response is activated and the secretion of antioxidants is increased to counter oxidative stress damage caused by renal ischemia-reperfusion.

### 3.6 Single-gene enrichment analysis of hub genes (GSEA)

**Figure 6:**
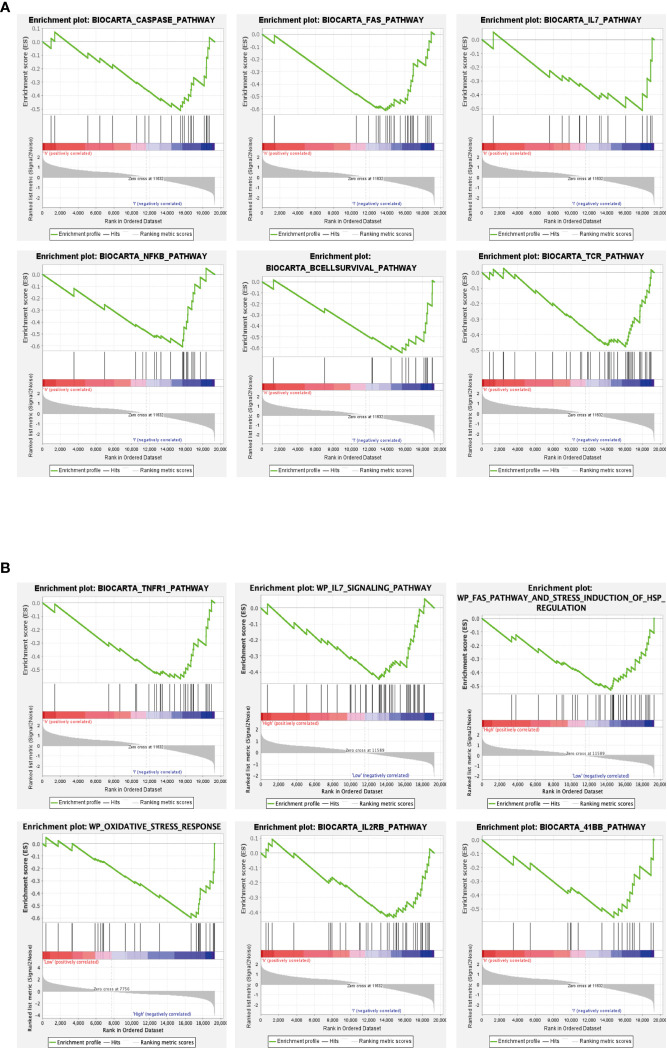
GSEA of Hub genes. **(A)** represents the signaling pathway associated with the GPX3 gene; **(B)** represents the signaling pathway associated with the GSTT1 gene.

We further explored the functions of the Hub genes GPX3 and GSTT1 in RIRIs using GSEA enrichment analysis. The caspase signaling pathway, FAS signaling pathway, IL-7 signaling pathway, NF-κB signaling pathway, B cell survival signaling pathway and TCR signaling pathway were negatively correlated with GPX3 gene expression (**Figure 6A**). TNFR1 signaling pathway, FAS signaling pathway, IL-7 signaling pathway, oxidative stress signaling pathway, IL2RB signaling pathway and 41BB signaling pathway were negatively correlated with GSTT1 gene expression (**Figure 6B**). Through further analyses of these pathways, we found that both IL-7 and NF-κB signaling pathway were key pathways for inducing oxidative stress in tissues. Both the caspase and FAS pathway were key pathways for apoptosis in tissue cells; the 41BB, TCR and IL2RB pathway were closely related to T cell activation and T-cell-mediated immune responses. The B cell survival signaling pathway was related to B-cell-mediated immune responses. Therefore, we believe that there is a strong relationship between the Hub genes GPX3 and GSTT1 and oxidative stress, apoptosis as well as immune responses during RIRIs.

### 3.7 Immune cell infiltration and correlation analysis of hub genes and immune cells

**Figure 7:**
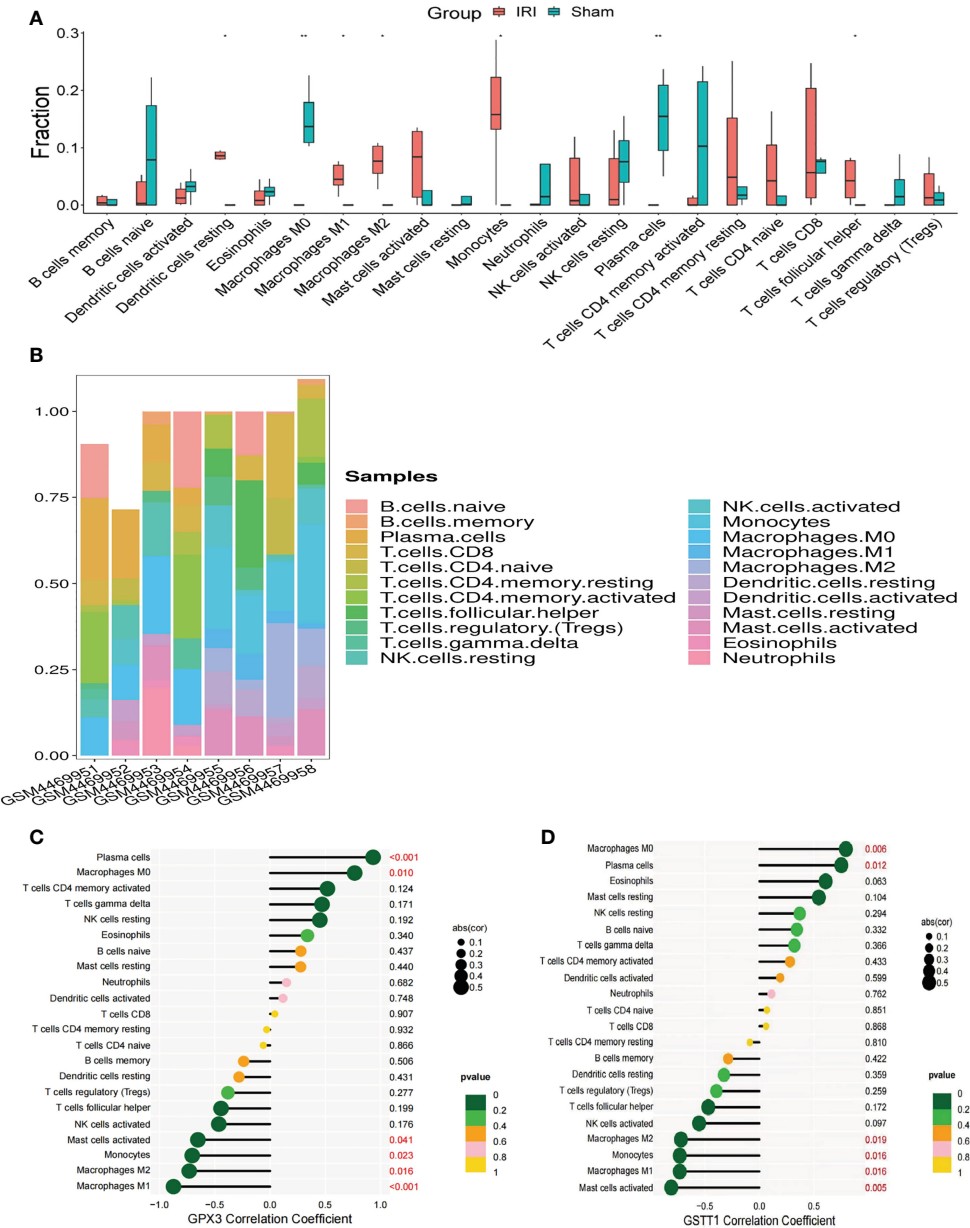
Immune cell infiltration and correlation analysis of Hub genes with immune cells. **(A)** represents the expression of 22 types of immune cells in the training set GSE148420 of the IRI and Sham group; **(B)** represents a stacked plot of the expression of 22 types of immune cells in each sample; **(C)** represents the correlation analysis plot between GPX3 and 22 types of immune cells; **(D)** represents the correlation analysis plot between GSTT1 and 22 types of immune cells. ** represents P<0.01, * represents P<0.05.

In GO and KEGG enrichment analyses of the above DEOSGs, immune cells also appeared to play a key role in the RIRI process. At the same time, in the single-gene GSEA enrichment analysis of GPX3 and GSTT1, a close relationship was also found between GPX3 and GSTT1 and immune-related signaling pathways. To further confirm the role of immune cells, after an immunoinfiltration analysis, we found significant differences in 7 immune cells of the IRI and Sham group (P<0.05), namely: dendritic cells, macrophages M0, M1 and M2, monocytes, plasma cells, T cell follicular cells (**Figures 7A, B**). To further understand the role of Hub genes in immune infiltration, we used Spearman correlation analysis to determine whether they are associated with immune cell infiltration. A correlation analysis showed that genes GPX3 and GSTT1 were significantly positively correlated with plasma cells and macrophage M0, while negatively correlated with monocytes and macrophages M1 and M2 (**Figures 7C, D**).

### 3.8 Construction of a transcription factor-hub gene network and a miRNA-Hub gene network

**Figure 8:**
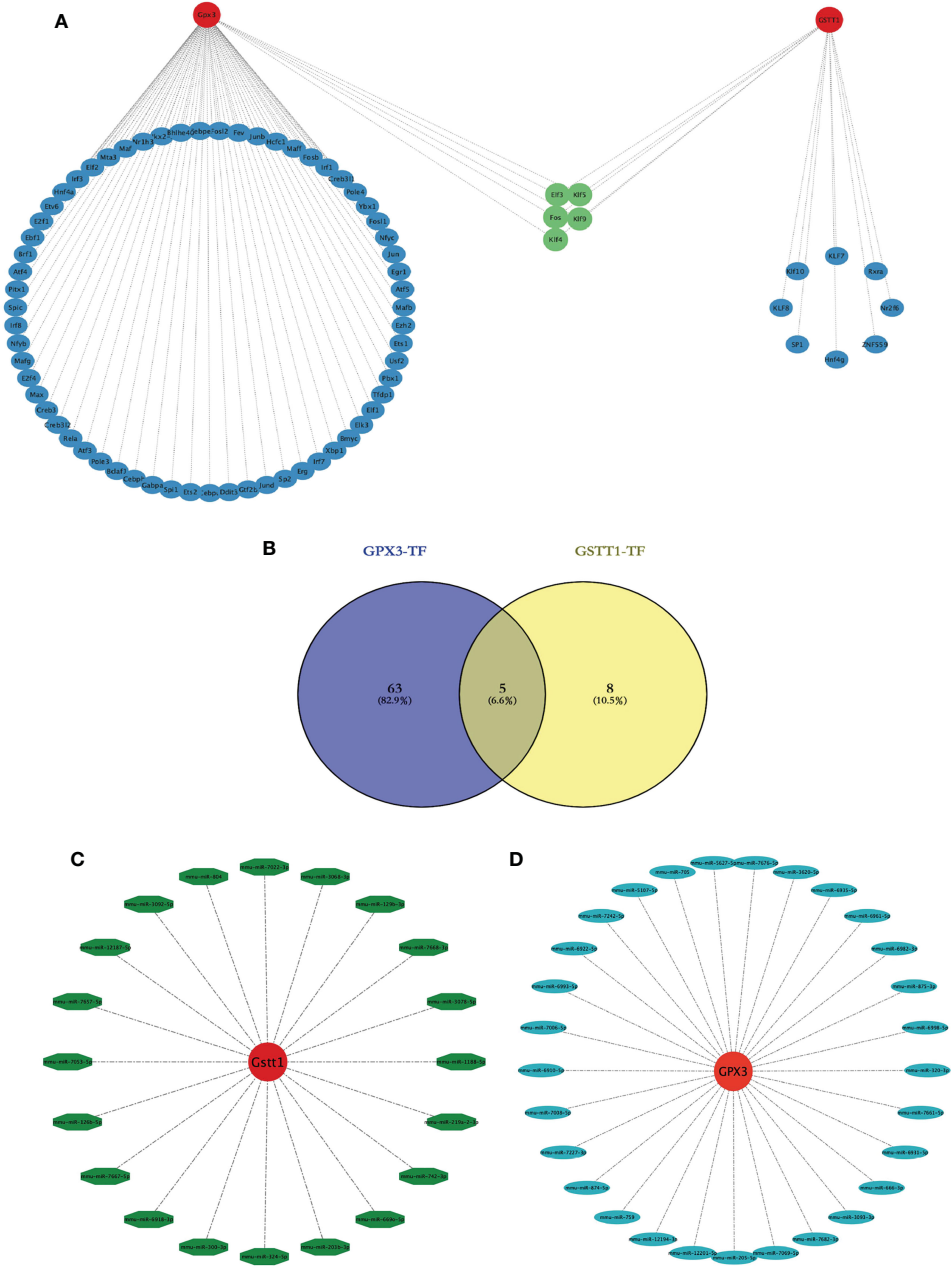
Construction of a transcription factor-Hub-gene network and a miRNA-Hub-gene network. **(A)** represents the Hub gene and TF regulatory network, red represents the Hub genes, blue represents TFs, and green represents TFs jointly expressed by two Hub genes. **(B)** represents the Venn diagram of the intersection of GPX3 and GSTT1 transcription factors; **(C)** represents the miRNA regulation map of GPX3; **(D)** represents the miRNA regulation map of GSTT1.

We wanted to better understand the role of Hub genes in the regulation of RIRIs. We predicted a total of 81 transcription factors (TFs) capable of interacting with 2 Hub genes using the Strust Database and constructed a TF-Hub gene regulatory network (**Figure 8A**); among them, there were 68 transcription factors that could regulate GPX3 and 13 that could regulate GSTT1. After an intersection of the two, we found that 5 TFs could regulate GPX3 and GSTT1 at the same time (**Figuress 8A, B**). We also predicted miRNAs capable of interacting with 2 Hub genes through the Starbase Database, constructing a miRNA-Hub-gene interaction network with a total of 29 miRNAs interacting with GPX3 (**Figure 8C**) and 20 interacting with GSTT1 (**Figure 8D**). The 2 Hub genes we screened had multiple binding sites for TFs and miRNAs, which could guide our further studies on their mechanism.

### 3.9 Identification of potential drugs for hub gene interactions

**Figure 9:**
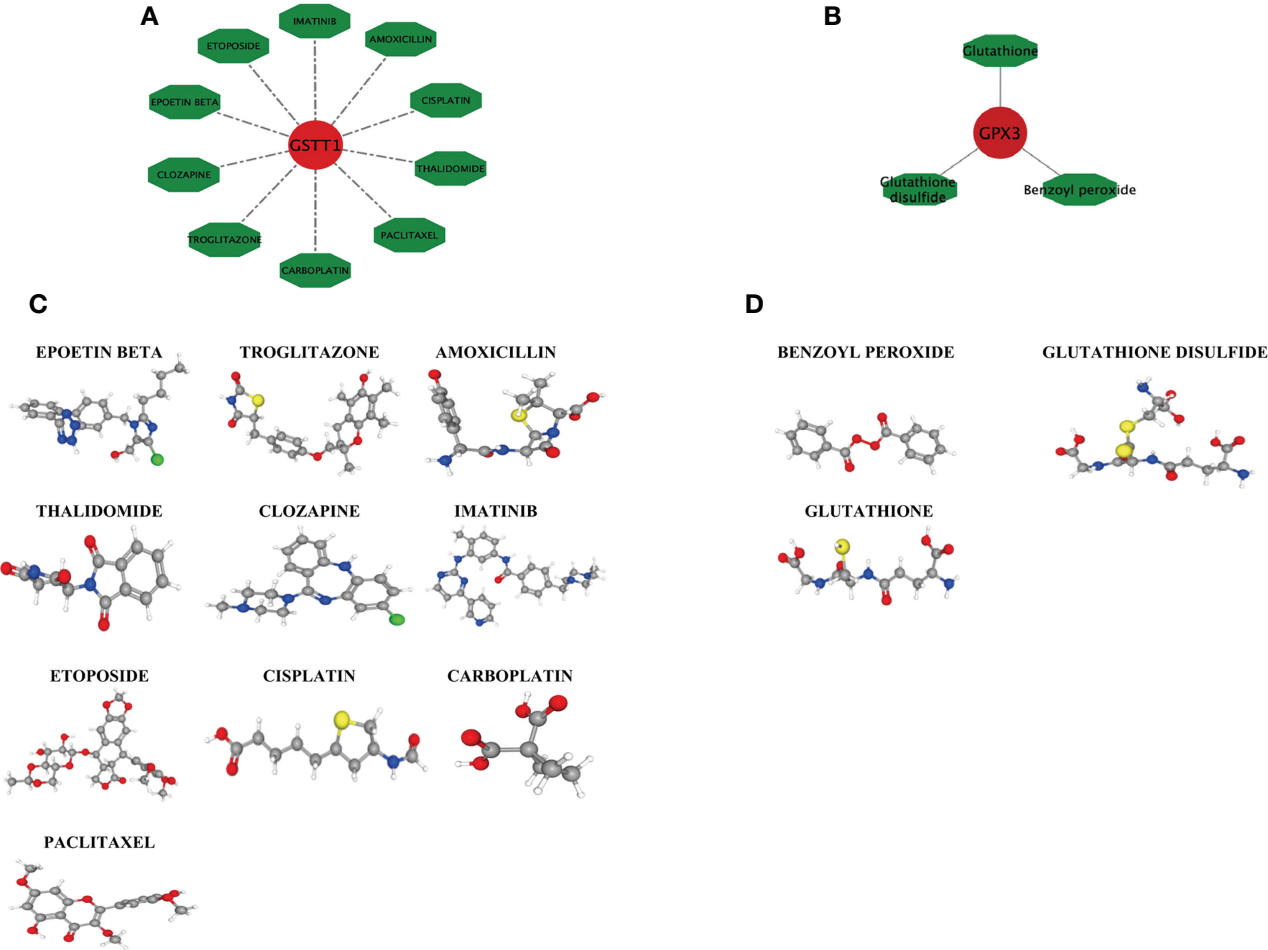
Drug interaction network with GSTT1 and GPX3 genes and related drug 3D structure diagram. **(A)** represents the action network diagram of GSTT1 gene-related drugs; **(B)** represents GPX3 gene-related drug action network diagram; **(C)** represents the 3D structure diagram of the GSTT1 gene-related drug; **(D)** represents a 3D structure diagram of GPX3 gene-related drugs.

Based on the DGIDB database, drugs or molecular compounds that might interact with the Hub genes were predicted, and a total of 13 drugs or molecular compounds that might have regulatory relationships with the Hub genes were screened, among which the number of drugs interacting with GSTT1 was the largest, and a total of 10 related drugs were identified. There were 3 drugs that interacted with GPX3 (**Figures 9A, B**). The 3D structure of the drugs is shown in **Figures 9C, D**, providing a theoretical basis for further research on Hub genes.

## 4 Discussion

RIRIs are one of the main causes of acute kidney injuries (AKIs), which can lead to a sharp decline in kidney functions and an increased mortality of severe cases (6). Therefore, early diagnosis, appropriate classification and early treatment play a crucial role in reducing mortality. Until now, the pathogenesis of RIRIs is very complex, which is not fully understood. Bioinformatic analysis allows us to understand the molecular mechanisms underlying the initiation and development of RIRIs, which can be used to explore potential targets for RIRI diagnosis and treatment. It has been found in current studies that a variety of physicochemical factors have a protective effect on RIRIs, mainly by inhibiting oxidative stress and reducing the release of inflammation-related factors (7). In addition, the activation of the immune system also appears to play a crucial role in RIRIs (11). Studying appropriate specific diagnostic markers and infiltrating immune cell identification can help restore prognosis for patients with RIRIs. In this study, bioinformatics was used to screen specific diagnostic molecular markers related to oxidative stress during RIRIs. Cibersort analysis helped to analyze the infiltration patterns of immune cells in diseases. We worked to identify diagnostic markers of RIRIs and explored the role of immune cell infiltration in RIRIs.

We downloaded the RIRI gene expression dataset from the GEO database, divided it into a training set and a validation set, and identified a total of 5456 DEGs under the differential expression threshold, of which 2486 showed an upregulation and 2970 showed a downregulation. To further clarify the genes associated with oxidative stress in DEGs, we obtained a total of 92 genes (OS) associated with oxidative stress from the Molecular Signatures Database. By intersecting with the DEGs described above, 30 intersecting genes were identified, which we defined as DEOSGs. In order to understand the potential biological functions of DEOSGs, we found through GO and KEGG enrichment analysis that these 30 DEOSGs were mainly enriched in glutathione metabolism and responses to oxidative stress. The glutathione peroxidase family (GPx) is considered one of the important selenoprotein families involved in the regulation of cellular redox balance. Tejchman et al. found that glutathione peroxidase (GPx) had oxidative stress effect during kidney transplantation and might have a protective effect on RIRIs (22). Moon et al. also proved this (23). Therefore, we believe that the metabolic process of glutathione plays a vital role in the RIRI process. At the same time, through GO and KEGG enrichment analysis, we observed that these 30 DEOSGs were also enriched in T cell activation and T cell receptor signaling pathways. Regulatory T cells play an important role in the inflammatory responses and the maintenance of immune balance. Xian et al. found that CXCR3 could alleviate RIRIs by increasing the expression of regulatory T cells (24). It can be seen that the role of immune cells in the process of RIRIs cannot be ignored.

In this study, we used two different screening methods (PPI protein interaction network and LASSO regression analysis) to select key genes, and the genes GPX3 and GSTT1 jointly screened through both methods were considered as the most diagnostically-valuable Hub genes in RIRIs. In the validation sets GSE58438 and GSE71647, the expression trend of GPX3 and GSTT1 in the Sham and IRI group was consistent with that in the training set, and there was statistical significance between their expression. At the same time, in the rat RIRI model, it was found through a Western Blot test of kidney tissues that the expression of proteins GPX3 and GSTT1 in the Sham group was significantly higher than that in the IRI group, which was consistent with the expression trend in the training and validation set, and the difference was statistically significant. At the same time, according to the immunohistochemical results of HE staining, PAS staining and renal tubular damage markers Kim-1 and NGAL, we found that compared with the Sham group, the degree of kidney tissue damage in rats in the IRI group was significantly aggravated. Furthermore, we analyzed the expression of oxidative stress markers HIF1-α and HO-1 using immunohistochemical. We found that the expression of HIF1-α and HO-1 in the IRI group was higher than that in the Sham group, which indicated that when the rat kidney suffered ischemia-reperfusion injury, the oxidative stress response was activated and the secretion of antioxidant substances increased to combat the oxidative stress damage caused by renal ischemia-reperfusion. These findings further support the role of oxidative stress in IRI. Therefore, GPX3 and GSTT1 have been verified and considered to be biomarkers related to oxidative stress during RIRIs. Based on previous studies, we have found that GPX3 belongs to the glutathione peroxidase family (GPXs), which is an important family of selenium-containing antioxidant enzymes in mammals (25). The biological functions of GPXs mainly depend on glutathione (GSH), which can be used as a thiol donor to reduce harmful substances such as hydrogen peroxide (H_2_0_2_) and lipid peroxide produced during the metabolism of human body, regulate the cellular microenvironment, and maintain the structural integrity as well as normal functions of cells (26). At the same time, the inflammatory responses can be reduced, and GPXs can exert an anti-inflammatory effect by regulating ROS (26). As a member of the glutathione S-transferase family, GSTT1 gene polymorphisms are currently closely related to tumor development and prognosis. However, Ghelli et al. (27) found that GSTT1 gene polymorphisms played a crucial role in the damage to oxidative stress in formaldehyde-induced oxidative stress. Through the above research, we believe that genes GPX3 and GSTT1 play an important role in the oxidative stress responses of tissues and organs, which may be potential targets for regulating oxidative stress during RIRIs.

In order to further explore the potential biological functions of GPX3 and GSTT1 in RIRIs, we found through GSEA that apoptosis-related signaling pathways (caspase signaling pathway, FAS signaling pathway), oxidative-stress-related signaling pathways (IL-7 signaling pathway, NF-κB signaling pathway) and immune-response-related signaling pathways (B cell survival signaling pathway, TCR signaling pathway, 41BB signaling pathway and IL2B signaling pathway) were negatively correlated with the gene expression of GPX3 and GSTT1. This means that in the RIRI group, the decrease in the level of GPX3 and GSTT1 may activate oxidative stress, apoptosis and immune responses, resulting in kidney tissue damage. Both in GO and KEGG enrichment assays and GSEA enrichment analyses on Hub genes, we found the role of immune responses in RIRIs. To further understand the role of immune cell infiltration in RIRIs and the correlation between Hub genes and immune cells, we used the Cibersort online tool to comprehensively evaluate immune infiltration in RIRIs. Compared with the Sham group, macrophage M1, macrophage M2 and monocytes in the IRI group were significantly upregulated. Macrophage M0 and plasma cells were significantly downregulated. In addition, a correlation analysis showed that genes GPX3 and GSTT1 were significantly positively correlated with plasma cells and macrophage M0, while negatively correlated with monocytes, and macrophages M1 and M2. The relationship between immune cells and acute ischemic kidney injuries has been elaborated by Zheng et al. They believe that in the process of acute kidney injury caused by ischemia involves the activation and recruitment of immune cells to the damaged kidney tissue. Various factors contribute to this process, such as increased expression of adhesion molecules, production of chemokines and cytokines, activation of the complement system, and increased permeability of renal vascular endothelium. Immune cells from both the innate and adaptive immune systems, including neutrophils, dendritic cells, macrophages, and lymphocytes, are believed to play a role in the pathogenesis of acute renal impairment caused by renal ischemia-reperfusion, with some subpopulations involved in the repair of injury (28), which further confirms that RIRIs are an inflammatory disease mediated by the immune system. Hu (29) et al. found in a study of RIRIs that the activation of macrophages M1 and M2 played a protective role in the process of RIRIs. Ai (30) et al. mentioned in the research process of renal fibrosis that by promoting the differentiation of macrophage M0 to macrophages M1 and M2, the process of renal fibrosis could be alleviated. It can be seen that the transformation process among macrophages M0, M1 and M2 may play an important role in the process of RIRIs. The infiltration and differentiation of monocytes into macrophages are a process in RIRIs that is thought to be the initial step in response to the inflammatory responses (31). In summary, immune cells also play a key role in the RIRI process. The pathophysiological mechanism of RIRIs and the formation of inflammatory responses may also involve the infiltration of immune cells, especially macrophages, and the specific mechanism needs to be further studied.

TFs are the main regulators in the biological process, which can regulate the expression of multiple gene targets and form a feedback loop. In the early stages of the diseases, many genes have already undergone significant changes, including many TFs. It is well known that in the development of RIRIs, the upregulation of many transcription factors drives the development process of RIRIs, some of which include FOS, JUN and P53 (32). This is consistent with our findings. In our study, according to our prediction, the Hub gene TFs may become new candidate genes for future research on the regulation of RIRI pathophysiological processes. The research on miRNA is a research hotspot in various fields, and its protective effect on RIRIs has attracted the attention of scholars. miR-125b-5p inhibits the expression of P53 in acute ischemic kidney injuries, thereby promoting tubular repair (33); miR-182-5p and miR-378a-3p regulate ferrozois caused by RIRIs (34); miR-30c-5p can regulate the transition from macrophage M1 to macrophage M2, thereby regulating changes in inflammatory factors and playing a protective role against RIRIs (35). However, the specific protective mechanism of miRNAs on RIRIs has not been fully elucidated, and its effect on RIRIs requires further research.

Finally, we predicted that 13 drugs or molecular compounds might be involved in the regulation of Hub genes, which could be a potential anti-RIRI drug. Many studies have confirmed the effect of drugs or molecular compounds on kidney IRIs. For example, propofol can enhance the polarization of macrophage M2 through the PPARγ/STAT3 signaling pathway and alleviate RIRIs (36); traprostalitib reduces mitochondrial damage during RIRIs in rats (37); dexmedetomidine may relieve RIRIs by suppressing the inflammatory responses (38). These drugs or molecular compounds may be potential drugs for the future treatment of RIRIs.

In this study, we used PPI-protein interaction networks and Lasso regression analysis to screen biomarkers related to oxidative stress in RIRIs. At the same time, we also used Cibersort to analyze immune cell infiltration in RIRIs, and systematically evaluated the role of oxidative stress as well as immune infiltration in RIRIs. However, we acknowledge that this study has some limitations. First of all, our analysis is the secondary mining of previously-released datasets, and different conclusions may be drawn due to different analysis ideas and perspectives. Secondly, the small number of samples used for bioinformatic analyses may reduce the accuracy of the results; therefore, large-sample-size experiments need to be considered in the future to confirm these findings. Thirdly, this study lacks relevant clinical information and self-sequencing results, which will be the direction of our future research on RIRIs. At the same time, we still need more experiments to prove the role and mechanism of GPX3 and GSTT1 in the process of RIRIs.

## 5 Conclusion

In summary, we performed a comprehensive bioinformatic analysis of the RIRI model and found that Hub genes such as GPX3 and GSTT1, as well as immune cell infiltration, might play a key role in the physiological and pathological processes of RIRIs. The analysis also revealed some TFs, miRNAs and drugs that might regulate Hub genes. The findings of this study may provide potential therapeutic targets and a deeper understanding of the mechanism of RIRIs, providing a new perspective for the diagnosis of RIRIs.

## Data availability statement

The datasets presented in this study can be found in online repositories. The names of the repository/repositories and accession number(s) can be found in the article/supplementary material.

## Ethics statement

The animal study was reviewed and approved by Animal Ethics Committee of the Children’s Hospital of Chongqing Medical University (IACUC Issue No: CHCMU-IACUC20220429002).

## Author contributions

JP, YH, and GW contributed to conception and design; GW and YH contributed to administrative support; XT, CY, and JL contributed to collection and processing of data; JP, XT, CY, and JL contributed to data analysis and interpretation; JP and JZ contributed to bioinformatics analysis; JP, XT, and JZ contributed to preparing all the figures. JP, JZ, and CY contributed to the experimental process. JP, JZ, and JL contributed to manuscript writing. All authors contributed to the article and approved the submitted version.
